# 1808. Burden of infectious diseases and bacterial antimicrobial resistance in China, 2019: a systematic analysis

**DOI:** 10.1093/ofid/ofad500.1637

**Published:** 2023-11-27

**Authors:** Chi Zhang, Yi-Qi Liu, Hong Zhao, Gui-Qiang Wang

**Affiliations:** Peking University First Hospital, Beijing, Beijing, China; Peking University First Hospital, Beijing, Beijing, China; Peking University First Hospital, Beijing, Beijing, China; Peking University First Hospital, Beijing, Beijing, China

## Abstract

**Background:**

Whether on a global scale or within China, the issue of bacterial antimicrobial resistance (AMR) has become a pressing concern. This study aimed to comprehensively investigate the disease burden of infectious diseases and AMR in China.

**Methods:**

Data on infectious diseases and AMR were collected by the Global Antimicrobial Resistance Burden study 2019 database. Our analysis steps consisted of 5 broad components: estimating the disease burdens of the total and 12 infectious syndromes by age and sex; describing the burden caused by 43 pathogens; calculating the burden caused by bacterial AMR in two scenarios (AMR associated deaths, AMR attributable deaths), respectively; estimating the AMR burden of 22 bacteria; calculating the burden of AMR by 22 bacteria and 19 antibiotics combinations.

**Results:**

In 2019, there were an estimated 1.3 million (95% UI 0.8-1.9) infection-related deaths in China, accounting for 12.1% of the total deaths for that year. Males were 1.5 times more affected than females. Bloodstream infections was the largest fatal burden syndrome, associating with 521392 deaths (286307-870583), followed by lower respiratory infections (373175 deaths), and peritoneal and intra-abdominal infections (152087 deaths). These five leading pathogens were *S aureus*, *A baumannii*, *E coli*, *S pneumoniae*, and *E spp.*, which were associated with 41.2% (502658/1218693) of all infection-related deaths. The pathogens of different infectious syndromes exhibit significant heterogeneity. In 2019, 602561 deaths were associated with bacterial AMR, including 145465 deaths attributable to bacterial AMR. Six leading pathogen-drug combinations were responsible for more than 0.5 million deaths in 2019: macrolides resistance *S aureus*, fourth-generation cephalosporins resistance *A baumannii*, carbapenems resistance *A baumannii*, third-generation cephalosporins resistance *A baumannii*, aminopenicillin resistance *E coli*, macrolides resistance *S pneumoniae*.

**Figure 1:**
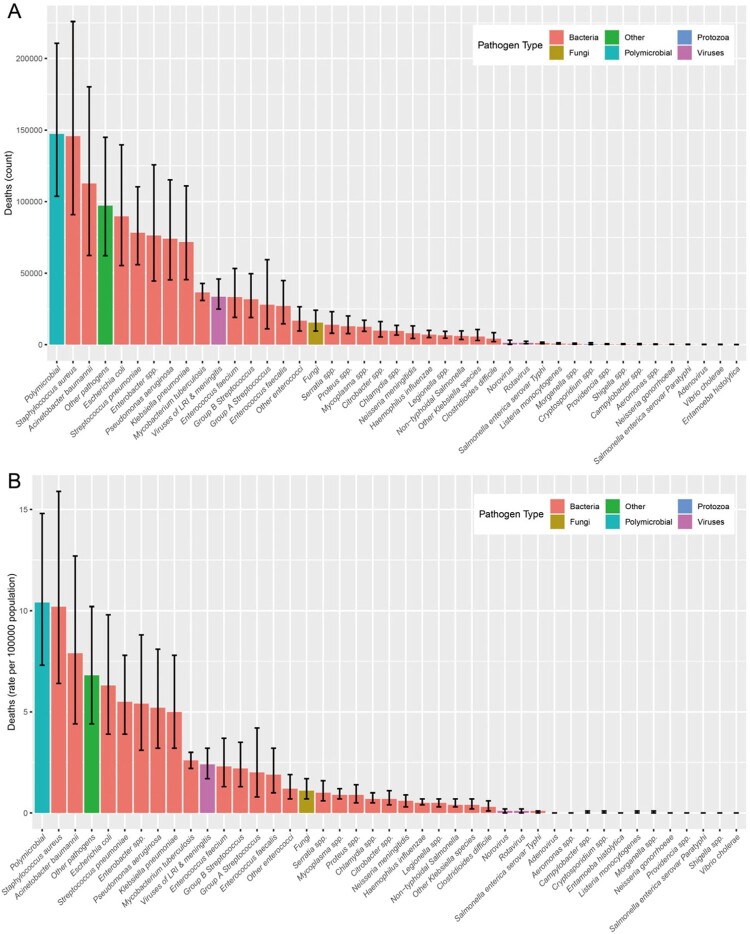
Number and rate of deaths by pathogens in China, 2019. (A) Number; (B) Rate per 100000 population

**Figure 2:**
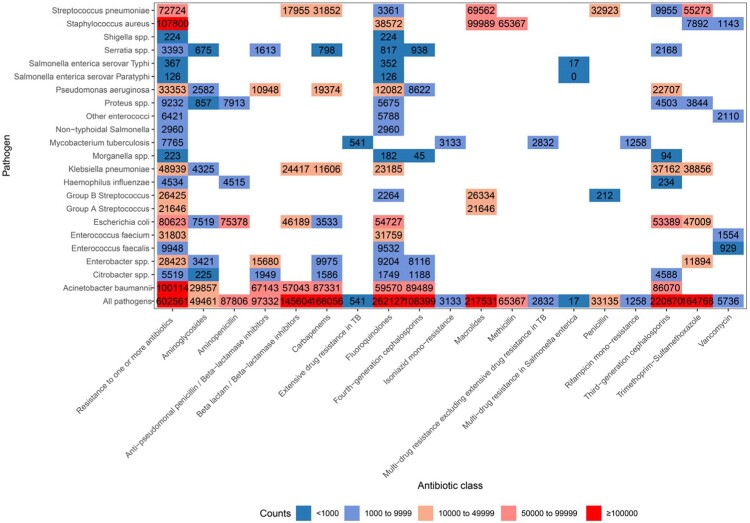
Number of deaths associated with bacterial antimicrobial resistance by pathogen-drug combination in China, 2019

**Conclusion:**

This study provides the comprehensive assessment of the burden of infectious diseases and bacterial AMR in China. The annual death toll of 1.3 million infection-related deaths, including 600000 deaths related to antibiotic resistance, requires sufficient attention.

**Disclosures:**

**All Authors**: No reported disclosures

